# Understanding the Behavioral Determinants of First Responder App Adoption by Integrating Perspectives From the Unified Theory of Acceptance and Use of Technology and Health Belief Model: Cross-Sectional Survey

**DOI:** 10.2196/69934

**Published:** 2025-09-09

**Authors:** Cas von Winckelmann, Robyn Vanherle, Lara Schreurs, Olivier Hoogmartens, Heidi Salaets, Jan De Spiegeleer, Marc Sabbe, Kathleen Beullens

**Affiliations:** 1 Media Psychology Lab Department of Communication Science KU Leuven Leuven Belgium; 2 Research Foundation - Flanders Brussels Belgium; 3 Leuven Institute for Healthcare Policy Department of Public Health and Primary Care KU Leuven Leuven Belgium; 4 Interpreting Studies Faculty of Arts Antwerp Campuses KU Leuven Antwerp Belgium; 5 Statistics and Risks Department of Mathematics KU Leuven Leuven Belgium; 6 Emergency Medicine Department of Public Health and Primary Care KU Leuven Leuven Belgium

**Keywords:** mHealth, mobile health, out-of-hospital cardiac arrest, first responder app, UTAUT, unified theory of acceptance and use of technology, health belief model, health technology adoption, cardiopulmonary resuscitation

## Abstract

**Background:**

Out-of-hospital cardiac arrests (OHCAs) are a leading cause of death worldwide, yet first responder apps can significantly improve outcomes by mobilizing citizens to perform cardiopulmonary resuscitation before professional help arrives. Despite their importance, limited research has examined the psychological and behavioral factors that influence individuals’ willingness to adopt these apps.

**Objective:**

Given that first responder app use involves elements of both technology adoption and preventive health behavior, it is essential to examine this behavior from multiple theoretical perspectives. Building on the unified theory of acceptance and use of technology (UTAUT) and health belief model (HBM), this study therefore developed an integrative framework to explain which behavioral determinants and demographic and health-related factors drive an individual’s willingness to install a first responder app for OHCA.

**Methods:**

We conducted a web-based cross-sectional survey (N=3660; mean age 49.95, SD 16.75 years; n=1909, 52.2% women) in June 2024 among Belgian adults. Behavioral determinants (UTAUT and HBM constructs), demographic (eg, age), and health-related (eg, cardiopulmonary resuscitation training experience) variables were measured using (multi-item) scales. Willingness to install the app served as the outcome variable. We developed a structural equation model using the *Lavaan* package in R and specified regression paths, on the one hand, between the behavioral determinants and willingness to install the app, and on the other hand, between the demographic and health-related factors and the behavioral determinants. Additionally, we conducted multiple group analyses to examine the moderating role of demographic and health-related factors on the relationships between the behavioral determinants and the willingness to install the app.

**Results:**

Our results revealed that 2 UTAUT variables (ie, facilitating conditions: β=.07; *P*=.003 and social influence: β=.16; *P*<.001) and 3 HBM variables (ie, perceived susceptibility: β=.06; *P*=.003, perceived barriers: β=–.29; *P*<.001, and perceived benefits: β=.38; *P*<.001) were associated with willingness to install a first responder app for OHCA. Additionally, most demographic and health-related factors were indirectly related to willingness via behavioral determinants, with age being the sole moderator. Specifically, a negative association between perceived severity and willingness was only observed among older adults. In addition, the positive relationship between perceived benefits and willingness was stronger for older adults compared to younger ones.

**Conclusions:**

Overall, the results of this study have both theoretical and practical implications. Theoretically, this study finds its relevance in extending the UTAUT and HBM to altruistic mobile health apps and advancing our understanding of technology adoption in health contexts. Practically, the study’s findings could inform real-life health campaigns aimed at enhancing citizen participation in first responder systems.

## Introduction

### Background

With the widespread use of mobile phones, the potential of technology to improve health outcomes has significantly increased [1]. Many people, for example, use their mobile devices to install apps for medical and public health practices, better known as mobile health (mHealth) [2]. Most of these apps enable users to monitor their health by, for instance, tracking their steps or creating personalized diet plans [3,4]. Additionally, besides promoting self-tracking, some mHealth apps can also be used to enhance the health of others and benefit society overall. Examples of such altruistic mHealth apps include contact tracing apps (CTAs), used during the recent COVID-19 pandemic [5], and first responder apps.

First responder apps alert and mobilize trained citizens to provide first aid in emergency situations, such as experiencing an out-of-hospital cardiac arrest (OHCA) [6]. Experiencing an OHCA is a leading cause of death worldwide [7], and in order to tackle this public health problem, early recognition of an OHCA and responding quickly to the situation are crucial. The use of a first responder app could thus be critical in this case, as citizens can be alerted to provide cardiopulmonary resuscitation (CPR) before the arrival of professional medical help, thereby ensuring a higher chance of survival [6,8]. However, despite the significant value of these apps, little is still known about their integration within societies for 2 reasons. First, these apps offer health benefits for others but not directly for users themselves, thereby leaving a gap in our understanding of users’ motivations behind adopting these altruistic apps. Second, first responder apps for OHCA differ substantially from other mHealth apps, as they are not designed for frequent use. Once installed, users are only required to engage with the app when they receive a notification requesting assistance for someone experiencing a cardiac arrest. As such, given this infrequent use, it is crucial to understand individuals’ willingness to install first responder apps for OHCA in order to promote the adoption of such apps in society.

Therefore, this study aims to contribute to the literature by defining which behavioral and individual factors play a role in citizens’ willingness to install a first responder app for OHCA. Given that first responder app use involves elements of both technology adoption and preventive health behavior, it is essential to examine this behavior from multiple theoretical perspectives, including the unified theory of acceptance and use of technology (UTAUT) [9] and the health belief model (HBM) [10]. Based on these theories, we will develop an integrated framework to explain the behavioral determinants that drive individuals’ willingness to install altruistic mHealth apps, that is, first responder apps for OHCA. Additionally, demographic and health variables will be examined to provide a comprehensive understanding of factors driving adoption willingness.

### Technology Perspective: UTAUT

To address the technology-related aspect of first responder app adoption, the study builds on the UTAUT model [9], a commonly used theoretical model for predicting the adoption of new technologies. The model consists of 4 key behavioral determinants of technology adoption: performance expectancy (ie, perceived effectiveness of the technology), effort expectancy (ie, the degree of ease associated with using the technology), social influence (ie, descriptive and injunctive norms), and facilitating conditions (ie, resources available that make it easier to adopt the new technology, such as internet access and owning a smartphone).

Given that this model integrates elements from various models and theories related to technology acceptance, it has been used frequently in the past to explain the adoption of apps that require frequent and consistent interaction, such as mobile banking apps [11], health monitoring apps [12,13], or travel apps [14]. Moreover, besides these frequently used apps, UTAUT has also been successful in explaining the adoption of CTAs [5,15-18]. Studies, for example, found that users were more inclined to install CTAs when they perceived the app as effective [5,17,18] and easy to use [18]. Additionally, social influence [16] and facilitating conditions, like smartphone ownership [19], were crucial predictors of CTA adoption. The study by Walrave et al [19], for example, showed that not owning a smartphone was one of the main reasons for not using a CTA.

These studies on CTAs, in turn, offer a relevant comparison for our study, as these apps, similar to first responder apps for OHCA, benefit public health and involve passive use (ie, only interacting when receiving a notification). Based on their insights, it is thus reasonable to assume that the factors outlined in the UTAUT model could also serve as effective predictors for the willingness to install a first responder app for OHCA—an app that is characterized by its altruistic nature, focus on immediate action, and infrequent use. In line with the prototype willingness model [20], we focus on willingness to install a first responder app rather than behavioral intention or actual use, since no such app currently exists in Belgium, thus making those measures less feasible at this stage. Building on the UTAUT model, this study therefore extends the literature by forming the following hypotheses:

- Hypothesis 1—Performance expectancy is positively associated with the willingness to install a first responder app for OHCA.
- Hypothesis 2—Effort expectancy is negatively associated with the willingness to install a first responder app for OHCA.
- Hypothesis 3—Facilitating conditions are positively associated with the willingness to install a first responder app for OHCA.
- Hypothesis 4—Social influence is positively associated with the willingness to install a first responder app for OHCA.

### Health Perspective: HBM

When installing a first responder app, individuals are not merely installing new software but are committing to potentially performing life-saving actions, that is, providing CPR to someone experiencing a cardiac arrest. Relying solely on a technological perspective, such as the UTAUT model, would therefore be insufficient to fully understand an individual’s willingness to install a first responder app for OHCA. Logically, this decision requires consideration of the health-related implications and responsibilities associated with the app’s use. Therefore, in addition to examining first responder app adoption through the lens of technology acceptance, it is essential to apply a health behavior change model to better understand the factors influencing the adoption of a first responder app for OHCA.

To do so, we will integrate the HBM [21], which is a widely recognized framework for understanding health-related behavior. The HBM suggests that individuals’ decisions to take health actions are influenced by 5 different factors: perceived susceptibility (ie, belief in the risk of experiencing a health problem), perceived severity (ie, belief in the seriousness of the health problem), self-efficacy (ie, confidence in performing the health behavior), perceived benefits (ie, factors that facilitate the health behavior), and perceived barriers (ie, factors that hinder the health behavior). In this context, a perceived benefit might be personal relevance [22], while a potential barrier could include privacy concerns [23]. Self-efficacy was later added as the fifth variable [21].

The model is often used to explain health behaviors aimed at protecting or improving one’s own health, such as condom use [24], or using mobile apps for managing chronic diseases [25]. However, limited research has explored the effectiveness of the HBM in predicting altruistic health behavior like providing CPR. Unlike self-beneficial behaviors, installing a first responder app, and thus committing oneself to potentially performing CPR, is a health behavior that only benefits another individual (ie, person experiencing a cardiac arrest). Several studies have examined the application of the HBM in the context of CTA adoption, which can also be considered an altruistic health behavior, as its primary goal is to minimize the spread of COVID-19. These studies indicate that self-efficacy, perceived barriers, and perceived benefits are the most significant predictors of adoption [18,26]. For instance, participants were more inclined to install the app when they felt confident in using it, recognized its benefits, and perceived fewer barriers to its adoption. While perceived severity and susceptibility have not consistently predicted adoption in studies on CTAs, they have shown positive associations with preventive health behaviors, such as mask-wearing [27].

Despite these insights related to CTAs, research on first responder apps and the determinants outlined in the HBM is limited. Still, building on findings from previous studies, the HBM likely also applies to the adoption of first responder apps for OHCA. For instance, as these apps can prevent severe outcomes, such as death or brain injury [28], individuals who perceive OHCA as a severe health risk may be more willing to install the app to be able to prevent such serious consequences. In addition, perceived susceptibility may influence willingness by heightening awareness of the app’s necessity in addressing a widespread health risk in society. Furthermore, because of the similar altruistic and preventive nature of CTAs, we can expect that self-efficacy and perceived benefits may positively influence the willingness to install a first responder app, while perceived barriers may negatively influence it.

Building on these insights, this study therefore aims to provide an extensive overview of how the variables of the HBM can explain the willingness to install a first responder app for OHCA. Based on current literature on mHealth apps and preventive health behavior, we propose the following hypotheses:

- Hypothesis 5—Self-efficacy is positively associated with the willingness to install a first responder app for OHCA.
- Hypothesis 6—Perceived susceptibility is positively associated with the willingness to install a first responder app for OHCA.
- Hypothesis 7—Perceived severity is positively associated with the willingness to install a first responder app for OHCA.
- Hypothesis 8—Perceived barriers are negatively associated with the willingness to install a first responder app for OHCA.
- Hypothesis 9—Perceived benefits are positively associated with the willingness to install a first responder app for OHCA.

### Influential Factors

Apart from the previously mentioned direct determinants of first responder app adoption, the HBM also identified a number of indirect factors affecting health behavior, including demographic factors and prior contact with the health problem [10]. Other health behavior change models such as the theory of planned behavior [29] and social cognitive theory [30] also emphasize the importance of demographic (eg, age, gender, and socioeconomic status [SES]) and social context variables in shaping individuals’ beliefs, attitudes, and social norms. Moreover, these theoretical propositions have also been confirmed in empirical research as, for example, older people tend to perceive health issues, such as COVID-19, as more severe [31].

Along with demographic variables, practicing a medical profession may also play a significant role in installing a first responder app for OHCA. Individuals working in the medical sector could, for example, perceive greater benefits from using such an app, as it can improve patient outcomes through bystander CPR [6,8]. Consequently, their perceived barriers to installing the app and expectations regarding its effectiveness may also be shaped by their professional experience and familiarity with emergency medical care.

In addition, as installing a first responder app for OHCA is a health-related behavior, the general health status of an individual and other relevant health variables should also be taken into account. First, given that CPR training significantly enhances the willingness to provide CPR [32,33], we could assume that trained individuals will be more likely to install a first responder app for OHCA, potentially via various behavioral determinants such as increased self-efficacy and perceived benefits. Second, the HBM highlights the relevance of prior contact with a health problem as a factor that can increase perceived susceptibility and severity, thereby influencing behavior [10]. In this study, having a cardiovascular disease and, therefore, an increased change of experiencing a cardiac arrest can be a relevant factor. Finally, indirect contact with health issues, such as having a family member or close acquaintance with a cardiovascular disease, could also affect individuals’ perceptions and decisions to adopt health-related behaviors like installing a first responder app for OHCA.

Since relationships between these influential factors (ie, demographic and health variables) and behavioral determinants of first responder app adoption have not been tested before, we pose the following research question (RQ):

- RQ1—Do demographic and health variables influence the behavioral determinants of willingness to install a first responder app for OHCA (performance expectancy, effort expectancy, social influence, facilitating conditions, self-efficacy, perceived severity, perceived susceptibility, perceived barriers, and perceived benefits)?

In addition to examining the variables mentioned earlier as predictors, it may be valuable to explore their potential moderating role. In the UTAUT model, gender and age already moderate the relationships between the key determinants and technology adoption [9,34]. For example, the relationship between performance expectancy and the intention to adopt a technology was stronger for men and young people, but the opposite was found for the other determinants [9,35]. Additionally, empirical research indicates that an individual’s education level moderates relationships between certain UTAUT variables (ie, performance expectancy and social influence) and individuals’ willingness to adopt new software [36]. This suggests that similar effects could be found for other demographic and health-related variables in the context of first responder OHCA apps. As there are again a limited amount of studies that investigated these moderations, we will examine the following RQ:

- RQ2—Do demographic and health variables moderate the relationships between the behavioral determinants (performance expectancy, effort expectancy, social influence, facilitating conditions, self-efficacy, perceived severity, perceived susceptibility, perceived barriers, and perceived benefits) and individuals’ willingness to install a first responder app for OHCA?

**Figure 1:**
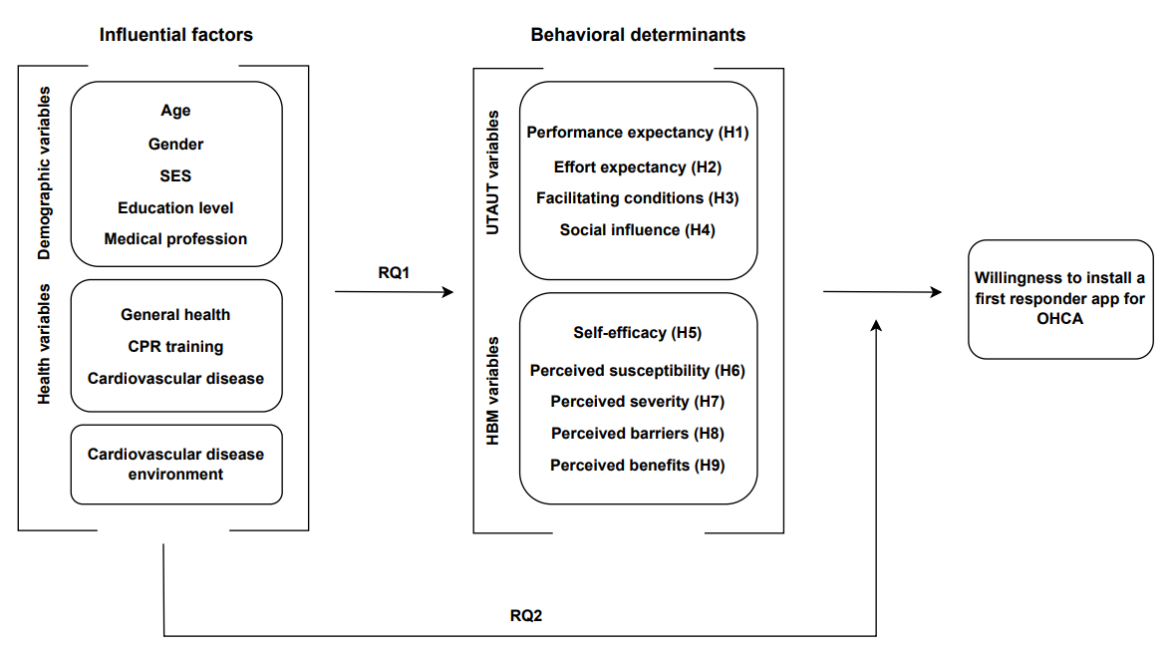
Conceptual framework of understanding and predicting willingness to install a first responder app for OHCA. Arrows indicate hypothesized associations based on theory and do not imply a causal relationship due to the cross-sectional design. CPR: cardiopulmonary resuscitation; H: hypothesis; HBM: health belief model; OHCA: out-of-hospital cardiac arrest; RQ: research question; SES: socioeconomic status; UTAUT: unified theory of acceptance and use of technology.

Overall, to fully understand the willingness to install a first responder app for OHCA, this study will evaluate a self-developed integrative framework that incorporates determinants from the UTAUT and HBM, along with additional influential factors that are particularly relevant to this context (Figure 1).

## Methods

### Study Design, Setting, and Participants

To test our hypotheses and address our RQs, we conducted a cross-sectional survey in June 2024 among a representative sample of Belgian adults. Participants were recruited out of a large national panel managed by a research agency, which ensured representativeness based on key sociodemographic characteristics, including gender, age, education level, and region of residence. Using quota sampling, the agency, with help from the researchers, actively monitored sample composition and adjusted invitations during data collection (eg, targeting underrepresented age groups). Eligible participants were individuals aged 18 years or older residing in Belgium. Respondents were asked to fill in a web-based Qualtrics questionnaire in Dutch or French, given the bilingual nature of the country.

### Ethical Considerations

The study received ethics approval from the Social and Societal Ethics Committee of the KU Leuven (G-2024-7825). At the beginning of the survey, participants were informed about the study’s objectives, procedures, their right to opt out at any time, and the confidentiality of their data, and were required to provide informed consent before continuing. Given the web-based procedure, the involved researchers were available via email to answer questions and solve practical and logistic issues. As compensation, participants received digital points from the research agency that recruited them, which could be redeemed for cash or donated to charity. To ensure privacy and confidentiality, all data were collected anonymously, with no personal identifiable information recorded, and were used solely for research purposes. In addition, the data were kept on a secure server.

### Study Size

Of the initial 3908 respondents, only those who completed more than the sociodemographic section of the questionnaire were included (n=3752). After removing 92 ineligible cases (underage, duplicate responses, or lack of consent), the final sample was 3660 participants, with a mean age of 49.95 (SD 16.75) years; 52.2% (n=1909) were women. The sample included 2205 Dutch-speaking and 1455 French-speaking respondents. Education levels varied, with 1.6% (n=58) having no formal education, 3.9% (n=144) primary, 36% (n=1319) secondary, 28.9% (n=1058) bachelor, 26.5% (n=971) master, and 3% (n=110) PhD. SES was assessed using the MacArthur Scale of Subjective Social Status [37]: 4% (n=149) low SES (scores 1-3), 70.3% (n=2576) medium SES (scores 4-7), and 25.5% (n=935) high SES (scores 8-10). Health-related data showed that 10.1% (n=369) worked as medical professionals, 58.8% (n=2153) had CPR training, 15.4% (n=564) had cardiovascular disease, and 30.4% (n=1113) knew someone with cardiovascular disease.

### Assessments and Data Sources

#### Demographic Variables

Respondents’ gender (1=man, 2=woman, and 3=other), year of birth, education level (1=no degree, 2=primary school degree, 3=secondary school degree, 4=bachelor degree, 5=master degree, and 6=PhD), and subjective SES were questioned [37]. Respondents were also asked if they practice a medical profession (1=yes and 0=no).

#### Health Variables

We asked respondents whether they had ever followed a CPR training (1=yes and 0=no) and whether they (1=yes and 0=no) or people in their surroundings (1=yes and 0=no) experienced a cardiovascular disease. Additionally, general health was measured with an adapted version of the Linear Analogue Scale [38]. Respondents had to indicate their general health condition on a scale from 1=worst to 100=best. On average, participants scored 71.11 (SD 18.05), suggesting that the overall health of our sample was good.

#### Willingness to Install a First Responder App for OHCA

To measure participants’ willingness to install a first responder app for OHCA, participants first received an explanation of what is meant with a first responder app. They were told that a first responder app for OHCA is a mobile app that alerts and mobilizes volunteer citizens to provide first aid to someone experiencing a cardiac arrest. Additionally, we explained the different features of the app (eg, location-based technology), the goal of the app, and that such an app is currently being set up in Belgium. Next, we asked respondents if they ever heard of a first responder app (n=458, 13.5% heard of a first responder app) and to indicate on a 5-point Likert scale (1=totally disagree to 5=totally agree) to what extent the following statement applied to them: “If a first responder app for OHCA is available in Belgium, I am willing to install it.” The mean score of 3.64 (SD 1.03) indicates a moderately high willingness to install the app.

#### Behavioral Determinants

##### UTAUT Determinants

###### Performance Expectancy

Based on the definition given by Venkatesh et al [9], performance expectancy was measured by a self-developed scale that asked respondents to indicate on a 5-point Likert scale (1=totally disagree to 5=totally agree) to what extent the following 2 statements applied to them: “This first responder app is an effective app for saving individuals experiencing a cardiac arrest” and “This first responder app would ensure that fewer people die from a cardiac arrest.” In these statements, this refers to the particular first responder app for OHCA in Belgium that is being set up. We found a high correlation between the 2 items (*r*=0.75; *P*<.001) and merged them together in 1 variable. On average, participants scored 3.90 (SD 0.78), indicating a strong positive perception of the app’s effectiveness.

###### Effort Expectancy

This was assessed using a 5-point Likert scale (1=totally disagree to 5=totally agree) item adapted from van der Waal et al [18], asking participants to rate the statement, “I think it will cost me a lot of time and energy to install this first responder app.” The average score was 2.06 (SD 1.05), indicating that most participants found the app easy to install.

###### Facilitating Conditions

Facilitating conditions were measured with a 5-point Likert scale (1=totally disagree to 5=totally agree) item based on van der Waal et al [18], asking participants to rate the statement, “I have a smartphone with internet access, allowing me to install this first responder app.” The mean score of 4.04 (SD 1.08) indicates high smartphone ownership and internet access among respondents.

###### Social Influence

Social influence was surveyed by measuring both injunctive and descriptive norms. We developed 2 items based on the definitions of Rimal and Real [39]. Injunctive norms were measured by asking respondents to indicate on a 5-point Likert scale (from 1=totally disagree to 5=totally agree) to what extent the following statement applied to them: “Most people in my environment would have a positive attitude toward this first responder app.” Descriptive norms were measured by: “Most people in my environment would install this first responder app, if this app would be available in Belgium,” and were evaluated on a 5-point Likert scale. Both norms were combined in social influences, as they were correlated (*r*=0.65; *P*<.001). With an average score of 3.28 (SD 0.78), the results indicate that social influences were moderately strong, meaning that the participants believed a fair number of people in their environment would install the first responder app and have a positive attitude toward it.

##### HBM Determinants

###### Self-Efficacy

A self-developed scale based on the definition of Bandura [30] asked respondents to indicate on a 5-point Likert scale (from 1=totally disagree to 5=totally agree) to what extent the following 2 statements applied to them: “It seems difficult to install this first responder app” and “I would be able to install this first responder app if I wanted to.” The first item was recoded for analysis and then merged with the second item into 1 construct, as there was a significant correlation between the 2 items (*r*=0.45; *P*<.001). Participants scored high on self-efficacy (mean 3.92, SD 0.87), suggesting that they feel confident in their ability to successfully install a first responder app.

###### Perceived Susceptibility of an OHCA

Perceived susceptibility was assessed on both personal and societal levels using self-developed items based on El-Toukhy [40]. Respondents rated their own risk (personal perceived susceptibility) and the average person’s risk (societal perceived susceptibility) of experiencing a cardiac arrest on a 5-point Likert scale (1=very low to 5=very high). Personal and societal susceptibility were correlated (*r*=0.56; *P*<.001) and combined into one measure with a mean of 2.90 (SD 0.64), indicating a moderate perceived susceptibility of an OHCA.

###### Perceived Severity of an OHCA

To measure respondents’ perceived severity of an OHCA on a personal and societal level, a self-developed scale adapted from El-Toukhy [40] asked them to indicate on a 5-point Likert scale (from 1=totally disagree to 5=totally agree) to what extent the following 2 statements applied to them: “I believe that if I experience a cardiac arrest, it could have serious consequences for me” and “I believe that if someone experiences a cardiac arrest, it can have serious consequences for that person.” The items, personal and societal perceived severity, respectively, correlated with each other (*r*=0.71; *P*<.001), and a mean score was calculated by averaging the 2 items. On average, participants perceived an OHCA as very severe (mean 4.45, SD 0.74).

###### Perceived Barriers

Based on technology and health behavior literature [19,21,41], we created 14 different items that reflected various barriers that respondents could experience while installing a first responder app (Multimedia Appendix 1). Examples of these barriers included phone-related issues, digital difficulties, privacy violations, and credibility of the app. Respondents had to indicate on a 5-point Likert scale (from 1=totally disagree to 5=totally agree) to what extent the statements applied to them (eg, “I would not install this first responder app because I am afraid my privacy will not be guaranteed.”). A manifest variable was created by summing the scores of all 14 items (range 14-70). Consequently, a low score on this variable indicates that a participant experiences few barriers, while a high score suggests that a participant encounters many barriers when it comes to installing a first responder app for OHCA. On average, participants scored 29.75 (SD 10.70), indicating that they generally did not feel substantially hindered in their ability to install the app.

###### Perceived Benefits

For perceived benefits, we developed 5 items that reflected various benefits respondents could perceive about installing a first responder app for OHCA (Multimedia Appendix 1), based on previous studies on altruistic health apps and providing CPR [22,32,42]. We measured benefits like personal relevance and altruistic satisfaction. Respondents had to indicate on a 5-point Likert scale (from 1=totally disagree to 5=totally agree) to what extent the statements applied to them (eg, “I would install this first responder app because it is my duty to help people.”). A manifest variable was formed by adding the scores from all 5 items, resulting in a possible range of 5 to 25. A lower score on this variable reflects fewer perceived benefits, while a higher score indicates more benefits related to installing a first responder app. The average participant score was 18.17 (SD 3.47), suggesting that most respondents saw the app as advantageous.

###### Data Analysis

After data cleaning, reliability indices (Multimedia Appendix 2), descriptive statistics (Multimedia Appendix 3), and zero-order correlations (Multimedia Appendix 3) were calculated. Then, we developed a structural equation model (SEM) using the *Lavaan* package in R (R Foundation for Statistical Computing) to test the hypotheses and address the RQs.

All variables with 2 items were entered as latent variables, while single-item variables were entered as manifest variables. Subsequently, we specified the regression paths, which were drawn between the behavioral determinants (ie, performance expectancy [hypothesis 1], effort expectancy [hypothesis 2], facilitating conditions [hypothesis 3], social influence [hypothesis 4], self-efficacy [hypothesis 5], perceived susceptibility [hypothesis 6], perceived severity [hypothesis 7], perceived barriers [hypothesis 8], and perceived benefits [hypothesis 9]; independent variables) and the willingness to install a first responder app for OHCA (ie, dependent variable) to answer our 9 hypotheses. Additionally, to examine RQ1, paths were specified between the behavioral determinants as dependent variables and the influential factors (ie, demographic and health-related variables) as independent variables.

To explore RQ2 and examine the moderating role of the influential factors on the relationships between the behavioral determinants and the willingness to install a first responder app for OHCA, multiple group analyses were conducted. We created groups based on the median for age, SES, general health, and education. For age, we compared younger and older participants; for SES, we compared low and high SES groups; for health, we compared poor and good health; and for education, we compared lower and higher educational attainment. Gender was recoded as a dichotomous variable, as the group of 11 participants who selected “other” was too small for meaningful analysis; these responses were therefore treated as missing values. The variables for medical profession, CPR training, cardiovascular disease, and cardiovascular disease in one’s environment were also dichotomous, meaning that participants were already divided into 2 distinct groups for each variable. The constrained model (ie, the model in which the paths of the behavioral determinants to willingness were specified to be the same across groups) was then compared with an unconstrained model (ie, the model in which the paths of the behavioral determinants to willingness were allowed to differ across groups), and a chi-square difference test was conducted to assess whether the model fit differed significantly between the groups.

The following indices were used to assess the model fit: chi-square/degrees of freedom, Comparative Fit Index, Tucker-Lewis Index, root mean square error of approximation (RMSEA), 90% CI for RMSEA, and standardized root mean square residual. The overall hypothesized model indicated an acceptable fit (*χ*^2^_95_=1230.8; *P*<.001; RMSEA=0.060; 90% CI 0.057-0.063; Comparative Fit Index=0.950; Tucker-Lewis Index=0.874; standardized root mean square residual=0.030). Although the chi-square (*df*) statistic and corresponding *P* value are reported, they were not used as the primary criterion for model acceptance or rejection, given that reliance on chi-square (*df*) alone can be overly restrictive in many research contexts. The study was preregistered on the Open Science Framework after data collection but before data analysis. The preregistration included the hypotheses and a detailed analysis plan (including model specifications, exclusion criteria, and handling of missing data). Additionally, an anonymized and cleaned version of the dataset and R code are available on Open Science Framework to enhance transparency and reproducibility of the analyses.

## Results

### Relations Between Behavioral Determinants and Willingness to Install a First Responder App for OHCA

Parameter estimates can be found in Table 1. First, regarding hypothesis 1 on the association between performance expectancy and willingness to install a first responder app for OHCA, we found no significant relationship between the 2 variables. Hypothesis 2 was also rejected, as we did not find a significant association between effort expectancy and willingness to install a first responder app for OHCA. For hypothesis 3, we found that more facilitating conditions were related to a higher willingness to install a first responder app for OHCA, which consequently confirms the hypothesis. In addition, social influence positively predicted willingness to install a first responder app for OHCA, thus confirming hypothesis 4. However, there were no significant associations between willingness to install a first responder app for OHCA, self-efficacy (hypothesis 5), and perceived severity (hypothesis 7), thus refuting hypothesis 5 and hypothesis 7. Perceived susceptibility on the other hand was positively related with the willingness to install a first responder app for OHCA, confirming hypothesis 6. Finally, the strongest associations with willingness to install a first responder app for OHCA occurred for perceived barriers (hypothesis 8) and perceived benefits (hypothesis 9), confirming hypothesis 8 and hypothesis 9.

**Table 1:** Parameter estimates of the structural equation model: relations between behavioral determinants and willingness to install a first responder app (FRA) for out-of-hospital cardiac arrest^a^.

Dependent variable and predictor			Estimate		SE		Standard estimate		*P* value
**Intention FRA**									
	Performance expectancy	0.036		0.031		0.026		.24	
	Effort expectancy	–0.044		0.049		–0.045		.36	
	Facilitating conditions	0.062^b^		0.021		0.065^b^		.003	
	Social influences	0.228^c^		0.029		0.157^c^		<.001	
	Self-efficacy	–0.118		0.102		–0.097		.24	
	Perceived susceptibility	0.126^b^		0.042		0.061^b^		.003	
	Perceived severity	–0.033		0.024		–0.023		.17	
	Perceived barriers	–0.028^c^		0.003		–0.292^c^		<.001	
	Perceived benefits	0.111^c^		0.005		0.375^c^		<.001	
	Age	–0.006^c^		0.001		–0.090^c^		<.001	
	Gender^d^	0.022		0.027		0.011		.41	
	SES^e^	–0.017		0.011		–0.024		.12	
	Education level	–0.013		0.015		–0.012		.42	
	Medical profession^f^	0.055		0.047		0.016		.24	
	General health	0.002		0.001		0.028		.07	
	Cardiovascular disease^f^	0.029		0.040		0.010		.47	
	CPR^g^ training^f^	–0.045		0.030		–0.021		.13	
	Cardiovascular disease in environment^f^	0.000		0.030		0.000		.99	

^a^*R*^2^ value is 0.446.

^b^*P*<.01.

^c^*P*<.001.

^d^Gender is coded: 1=woman, 2=man, and 3=other.

^e^SES: socioeconomic status.

^f^Medical profession, cardiovascular disease, CPR training, and cardiovascular disease in environment are coded: 1=yes and 0=no.

^g^CPR: cardiopulmonary resuscitation.

### Relations Between the Influential Factors and the Behavioral Determinants

To answer RQ1, we examined how demographic and health variables related to the behavioral determinants of willingness to install a first responder app for OHCA (see Tables 2 and 3 for all parameter estimates).

**Table 2:** Parameter estimates of the structural equation model: relations between demographic variables and the behavioral determinants^a^.

Dependent variable and predictor			Estimate		SE		Standard estimate		*P* value		*R* ^2^	
**Performance expectancy**												0.015
	Age	0.004^b^		0.001		0.093^b^		<.001				
	Gender	–0.028		0.028		–0.019		.32				
	SES^c^	0.007		0.011		0.014		.52				
	Education level	–0.013		0.015		–0.018		.39				
	Medical profession	–0.079		0.047		–0.032		.09				
**Effort expectancy**												0.095
	Age	0.013^b^		0.001		0.203^b^		<.001				
	Gender	–0.082		0.035		–0.040		.01				
	SES	–0.034		0.014		–0.048		.01				
	Education level	–0.099^b^		0.019		–0.095^b^		<.001				
	Medical profession	0.366^b^		0.059		0.106^b^		<.001				
**Facilitating conditions**												0.073
	Age	–0.012^b^		0.001		–0.190^b^		<.001				
	Gender	0.026		0.036		0.012		.48				
	SES	0.056^b^		0.015		0.076^b^		<.001				
	Education level	0.082^b^		0.020		0.076^b^		<.001				
	Medical profession	–0.167^d^		0.062		–0.047^d^		.007				
**Social influences**												0.015
	Age	–0.003^b^		0.001		–0.076^b^		<.001				
	Gender	0.007		0.027		0.005		.80				
	SES	0.034^d^		0.011		0.070^d^		.002				
	Education level	–0.015		0.015		–0.022		.30				
	Medical profession	0.078		0.047		0.033		.09				
**Self-efficacy**												0.191
	Age	–0.017^b^		0.001		–0.335^b^		<.001				
	Gender	0.085^d^		0.032		0.051^d^		.009				
	SES	0.028		0.013		0.049		.03				
	Education level	0.110^b^		0.018		0.131^b^		<.001				
	Medical profession	–0.358^b^		0.055		–0.129^b^		<.001				
**Perceived susceptibility**												0.428
	Age	0.018^b^		0.001		0.594^b^		<.001				
	Gender	0.018		0.014		0.018		.22				
	SES	–0.015^d^		0.006		–0.045^d^		.008				
	Education level	–0.057^b^		0.008		–0.115^b^		<.001				
	Medical profession	0.123^b^		0.025		0.075^b^		<.001				
**Perceived severity**												0.068
	Age	0.002		0.001		0.045		.02				
	Gender	–0.013		0.026		–0.009		.61				
	SES	0.007		0.010		0.014		.51				
	Education level	0.135^b^		0.014		0.195^b^		<.001				
	Medical profession	–0.248^b^		0.044		–0.108^b^		<.001				
**Perceived barriers**												0.053
	Age	–0.008		0.012		–0.012		.49				
	Gender	–1.018^d^		0.360		–0.049^d^		.005				
	SES	–0.377		0.146		–0.052		.01				
	Education level	–0.726^b^		0.199		–0.068^b^		<.001				
	Medical profession	2.882^b^		0.613		0.082^b^		<.001				
**Perceived benefits**												0.090
	Age	0.003		0.004		0.014		.43				
	Gender	0.158		0.115		0.023		.17				
	SES	0.007		0.046		0.003		.89				
	Education level	–0.115		0.063		–0.033		.07				
	Medical profession	0.786^b^		0.195		0.068^b^		<.001				

^a^Gender is coded: 1=woman, 2=man, and 3=other. Medical profession is coded: 1=yes and 0=no.

^b^*P*<.001.

^c^SES: socioeconomic status.

^d^*P*<.01.

**Table 3:** Parameter estimates of the structural equation model: relations between health variables and the behavioral determinants^a^.

Dependent variable and predictor		Estimate	SE	Standard estimate	*P* value	*R* ^2^	
**Performance expectancy**							0.015
	General health	0.003^b^	0.001	0.067^b^	.002		
	Cardiovascular disease	–0.013	0.041	–0.006	.75		
	CPR^c^ training	0.087^b^	0.029	0.058^b^	.003		
	Cardiovascular disease in environment	0.004	0.031	0.002	.90		
**Effort expectancy**							0.095
	General health	–0.002	0.001	–0.043	.02		
	Cardiovascular disease	0.098	0.052	0.034	.06		
	CPR training	–0.211^d^	0.036	–0.099^d^	<.001		
	Cardiovascular disease in environment	0.094	0.039	0.041	.02		
**Facilitating conditions**							0.073
	General health	0.003^b^	0.001	0.053^b^	.005		
	Cardiovascular disease	–0.018	0.054	–0.006	.73		
	CPR training	0.154^d^	0.038	0.070^d^	<.001		
	Cardiovascular disease in environment	0.025	0.041	0.011	.54		
**Social influences**							0.015
	General health	0.002	0.001	0.040	.07		
	Cardiovascular disease	0.013	0.041	0.007	.75		
	CPR training	–0.072	0.029	–0.050	.01		
	Cardiovascular disease in environment	0.000	0.031	0.000	.99		
**Self-efficacy**							0.191
	General health	0.003^b^	0.001	0.058^b^	.009		
	Cardiovascular disease	–0.071	0.049	–0.031	.14		
	CPR training	0.180^d^	0.034	0.105^d^	<.001		
	Cardiovascular disease in environment	–0.053	0.036	–0.029	.15		
**Perceived susceptibility**							0.428
	General health	–0.003^d^	0.000	–0.094^d^	<.001		
	Cardiovascular disease	0.038	0.022	0.027	.08		
	CPR training	–0.031	0.015	–0.030	.04		
	Cardiovascular disease in environment	0.056^b^	0.016	0.051^b^	.001		
**Perceived severity**							0.068
	General health	0.001	0.001	0.038	.07		
	Cardiovascular disease	0.052	0.038	0.027	.17		
	CPR training	0.179^d^	0.027	0.126^d^	<.001		
	Cardiovascular disease in environment	0.029	0.029	0.019	.31		
**Perceived barriers**							0.053
	General health	–0.061^d^	0.011	–0.103^d^	<.001		
	Cardiovascular disease	1.159	0.540	0.039	.03		
	CPR training	–2.325^d^	0.380	–0.107^d^	<.001		
	Cardiovascular disease in environment	0.676	0.405	0.029	.09		
**Perceived benefits**							0.090
	General health	0.014^d^	0.004	0.075^d^	<.001		
	Cardiovascular disease	–0.062	0.172	–0.007	.72		
	CPR training	1.818^d^	0.121	0.257^d^	<.001		
	Cardiovascular disease in environment	0.530^d^	0.129	0.070^d^	<.001		

^a^Cardiovascular disease, CPR training, and cardiovascular disease in environment are coded: 1=yes and 0=no.

^b^*P*<.01.

^c^CPR: cardiopulmonary resuscitation.

^d^*P*<.001.

#### Demographic Variables

The results showed that practicing a medical profession was positively related to effort expectancy, perceived susceptibility, perceived barriers, and benefits but negatively with facilitating conditions, self-efficacy, and perceived severity (Table 2). Age was positively associated with performance expectancy, effort expectancy, and perceived susceptibility but negatively with facilitating conditions, social influences, and self-efficacy. Furthermore, education level correlated with 6 of the 9 behavioral determinants: negatively with effort expectancy, perceived susceptibility, and perceived barriers and positively with facilitating conditions, self-efficacy, and perceived severity. Gender was only positively related to self-efficacy and negatively related to perceived barriers. This means that men had higher levels of self-efficacy and women perceived more barriers to install a first responder app for OHCA. Finally, we found positive relationships between SES and facilitating conditions and social influences, whereas SES was negatively correlated with perceived susceptibility (Table 2).

#### Health Variables

General health proved to be an important variable, as the higher participants rated their health, the higher they scored on performance expectancy, facilitating conditions, self-efficacy, and perceived benefits, but the lower they scored on perceived susceptibility and barriers. Moreover, having done a CPR training was also positively related to 5 behavioral determinants (ie, performance expectancy, facilitating conditions, self-efficacy, perceived severity, and perceived benefits) but negatively with effort expectancy and perceived barriers. Surprisingly, we found no significant associations between any of the behavioral determinants and experiencing a cardiovascular disease oneself. Knowing someone in your close environment who is experiencing a cardiovascular disease, on the other hand, increased perceived susceptibility and perceived benefits of a first responder app for OHCA (Table 3).

We also explored the indirect relationships between the influential factors and willingness to install the app through the behavioral determinants. Only a limited number of these relationships were significant. Multimedia Appendix 4 reports the estimates.

### The Moderating Role of the Influential Factors

Regarding RQ2, multiple group analyses were performed to examine potential moderating effects of the demographic and health variables (Table 4). Age was the only significant moderator in the SEM, with the chi-square difference test indicating a significant difference between the fits of the constrained and unconstrained model (Δ*χ*^2^_9_=23.690; *P*=.005). The post hoc tests revealed that age differences were only present for 2 relations within the model (Table 5). First, perceived severity was differently related to willingness to install a first responder app for OHCA. For young adults, there was no significant relation (β=.028; *P*=.49), and for older adults, there was a negative relationship (β=–.072; *P*=.03). Thus, for older people perceiving a cardiac arrest as severe related negatively to willingness to install the app. In addition, the association between perceived benefits and willingness to install a first responder app for OHCA was significantly stronger for older people (β=.123; *P*<.001) compared to younger people (β=.092; *P*<.001)⁠.

**Table 4:** Multiple group analyses.

Moderator	Δ*χ*^2^ (Δ*df*=9)	*P* value
Age	23.690^a^	.005
Gender	10.195	.34
SES^b^	7.478	.59
Education level	6.443	.70
Medical profession	11.397	.25
Health	8.091	.53
Cardiovascular disease	8.704	.47
CPR^c^ training	9.901	.36
Cardiovascular disease in environment	4.890	.84

^a^*P*<.01.

^b^SES: socioeconomic status.

^c^CPR: cardiopulmonary resuscitation.

**Table 5:** Post hoc chi-square difference tests for age.

	Δ*χ*^2^ (Δ*df*=1)	*P* value	β young	β old
Performance expectancy→intention to install FRA^a^	2.467	.17	—^b^	—
Effort expectancy→intention to install FRA	0.128	.72	—	—
Facilitating conditions→intention to install FRA	0.022	.88	—	—
Social influences→intention to install FRA	3.013	.08	—	—
Self-efficacy→intention to install FRA	0.885	.35	—	—
Perceived susceptibility→intention to install FRA	0.053	.82	—	—
Perceived severity→intention to install FRA	3.920^c^	.048	.028	–.072
Perceived barriers→intention to install FRA	0.161	.69	—	—
Perceived benefits→intention to install FRA	7.820^d^	.005	.092	.123

^a^FRA: first responder app.

^b^Not applicable.

^c^*P*<.05.

^d^*P*<.01.

## Discussion

### Overview

OHCAs are one of the leading causes of death worldwide, impacting approximately 350,000 people annually in Europe [7]. With survival rates ranging from 5% to 25% [7], first responder apps for OHCAs offer a vital solution to increase survival rates by mobilizing volunteer citizens to provide CPR in emergency situations. However, despite the emergence of these apps, little is still known about their adoption processes. Building on a comprehensive framework that integrates the UTAUT model, the HBM, and other contextual variables, this study therefore contributes to the literature by examining which factors influence citizens’ willingness to install a first responder app for OHCA. While this framework suggests meaningful relationships between the variables studied, it is important to note that these are correlational and do not imply causality due to the cross-sectional nature of the study.

### Main Findings Related to Hypotheses

This study offers partial evidence for the use of UTAUT determinants in understanding the adoption of first responder apps for OHCA, expanding our insights into how the model applies to altruistic mHealth apps. Specifically, only facilitating conditions (hypothesis 3) and social influence (hypothesis 4) were positively related to willingness to install a first responder app for OHCA, whereas performance and effort expectancy were not. First, aligning with facilitating conditions in studies on CTAs [18,19], people with internet and smartphone access were more willing to install a first responder app for OHCA, which is unsurprising given that these conditions are essential for installing and using a first responder app [19]. In addition, individuals who perceived more positive descriptive and injunctive norms were more willing to install a first responder app for OHCA. This aligns with a systematic review on CTAs showing that social influence is one of the most significant predictors of novel app adoption [16]. Moreover, research shows that social influence is a crucial predictor of the intention to perform bystander CPR [43], which may also help clarify the relationship observed in this study.

Performance (hypothesis 1) and effort expectancy (hypothesis 2) were not significantly associated with willingness to install a first responder app for OHCA. While previous studies on CTAs and other health apps have reported mixed results for effort expectancy [5,18,44], performance expectancy has been classified as a strong predictor [13,15,17,18]. These insignificant findings in this study could be attributed to the fact that the first responder app has not yet been implemented in Belgium, thus making it hard for individuals to estimate the app’s effectiveness (performance expectancy) and ease of use (effort expectancy) based solely on the brief description provided.

Regarding the HBM determinants, we identified 3 significant relationships (ie, perceived susceptibility, barriers, and benefits) with willingness to install a first responder app for OHCA. First, even though mixed findings occurred for perceived susceptibility in mHealth app literature [18,25,45], this variable was a significant predictor for the willingness to install a first responder app (hypothesis 6). This indicates that individuals who perceive themselves to be at a higher risk of experiencing a cardiac arrest are more inclined to adopt the app. Consequently, their personal risk assessment likely influences their perception of the app’s importance in society. In addition, perceived barriers (hypothesis 8) and benefits (hypothesis 9) were significantly associated with willingness to install a first responder app for OHCA, with barriers negatively and benefits positively relating to willingness. In other words, the more barriers participants perceived, the less likely they were to install the app, while those who saw more benefits were more inclined to install. These findings align with previous research on health app adoption and bystander CPR, where barriers such as usability issues or privacy concerns [19,21,22,41], and perceived benefits like personal relevance and altruistic satisfaction [21,31,42], have been shown to be key predictors. Interestingly, perceived barriers and benefits emerged as the strongest predictors, highlighting their critical role in users’ willingness to install a first responder app for OHCA.

Finally, there was no significant relationship between self-efficacy (hypothesis 5) or perceived severity (hypothesis 7) and willingness to install a first responder app for OHCA. The absence of a link with self-efficacy is surprising, as it contradicts studies on CTAs [18,26] and other mHealth technologies [46,47], where self-efficacy often emerged as a strong predictor, sometimes even the strongest. One possible explanation could lie in the difference between technology-related and health-related self-efficacy. In health research, self-efficacy often involves confidence in performing complex behaviors, like CPR. As such, it may not be self-efficacy related to feeling able to install the app, which can be fairly simple, but rather self-efficacy related to feeling capable of providing CPR if alerted, which may predict individuals’ willingness to install the app. This study only measured technology-related self-efficacy, which might explain the absence of a significant link in the findings. Future studies should explicitly differentiate between technology-related and health-related self-efficacy to clarify their respective roles in shaping willingness to adopt first responder apps. Additionally, mixed results regarding perceived severity have been reported in the literature [18,48], suggesting variability in its influence across different health behaviors. In the context of first responder apps for OHCA, the lack of a significant relationship might be explained by the psychological and physical distance associated with such emergencies. Although citizens recognize that OHCA is a severe, life-threatening condition, they may perceive it as happening outside of their immediate, familiar environment and therefore do not feel a personal sense of responsibility. This feeling of detachment might lessen their willingness to install a first responder app.

### Main Findings Related to RQs

We formulated 2 RQs to explore influential factors that may affect the behavioral determinants of willingness to install a first responder app for OHCA and to examine the potential moderating effects of these factors on the relationships between the determinants and willingness. As expected, age proved to be a significant variable for 2 main reasons. First, it was associated with 6 of the 9 behavioral determinants. Second, it was the only moderating factor in the model. More specifically, there was an age difference in the relation between perceived severity and willingness: no significant link was found for adults younger than 50 years of age, while a negative association was observed for those older than 50 years. Older adults who viewed cardiac arrest as severe were thus less willing to install a first responder app for OHCA. This finding is surprising; yet, it may suggest that older adults, who often have a more skeptical attitude toward technology [49], may associate severe medical emergencies with the need for professional intervention rather than assistance from lay responders. This perception could reduce their confidence in the app’s usefulness or appropriateness. Alternatively, they may feel less personally capable or comfortable engaging with the app, especially in high-stakes situations. While these interpretations remain speculative, they highlight the need for further research into how age-related attitudes toward technology and emergency care shape the willingness to install health apps. Additionally, there was an age difference in the relationship between perceived benefits and willingness, with a stronger positive relation identified among older adults. The stronger positive relationship may indicate that older people, unlike digital natives, require clearer and more substantial advantages before they are willing to adopt new technologies. This aligns with Rogers’ diffusion of innovations theory [50], which suggests that individuals assess the relative advantages and disadvantages of an innovation before deciding to adopt it. Moreover, it is in line with the UTAUT model that says that all relationships except for performance expectancy are stronger for older people [9,35].

In accordance with the HBM [10], also other demographic variables such as gender, SES, and education level were related to several of the behavioral determinants, and in that way indirectly to willingness to install a first responder app for OHCA. For example, younger people and men experienced more self-efficacy, which aligns with existing research on technology-related self-efficacy [51,52], and people with a lower SES perceived themselves and their environment as more susceptible to a cardiac arrest. This relationship is logical, as general health showed a strong correlation with SES.

Health variables, although often not included within behavior change models [10,29], seemed to play a meaningful role. First of all, medical professionals perceived greater benefits of a first responder app for OHCA than those outside the medical field, which aligns with expectations, given the app’s potential to improve patient outcomes [6]. However, they also reported more barriers, lower self-efficacy, higher effort expectancy, and lower scores on facilitating conditions and perceived severity. This pattern of results, where medical professionals score lower on multiple variables associated with willingness to install the app, may be attributed to several factors. First, they may be less technology-savvy or more resistant to health care digitalization, a trend supported by previous research [53]. Second, their familiarity with the consequences of a cardiac arrest could reduce the perceived severity, as the nature of their work may lead them to view a cardiac arrest as a more routine, manageable event. Third, they may have reservations about the general public’s ability to provide effective CPR, leading to greater skepticism toward the app’s effectiveness in nonprofessional hands. This skepticism may be unfounded, as individuals trained in CPR scored higher across 7 of the 9 behavioral determinants in this study, highlighting the potential benefits of promoting CPR training among the general public in first responder app adoption.

Furthermore, the general health status of individuals affected the majority of the behavioral determinants, but surprisingly, this was not the case for having cardiovascular disease. This is in contrast with the HBM, which states that prior experience or direct contact with a health issue typically influences perceptions and attitudes toward related health behaviors [10]. Indeed, one would assume that individuals with a cardiovascular disease, given their increased susceptibility to a cardiac arrest, would perceive themselves as more at risk and potentially recognize greater benefits in a first responder app for OHCA. Although we did find individual correlations between having a cardiovascular disease and several determinants (eg, positively related to effort expectancy, perceived susceptibility, and perceived barriers), their significance diminished when tested within the full model. Given the cross-sectional nature of our study, making it impossible to make claims about directionality, longitudinal research is needed to further clarify these correlations. On the contrary, knowing people in your environment who have a cardiovascular disease heightened individuals’ awareness of personal risk and ensured more perceived benefits of a first responder app for OHCA.

### Limitations

This study has several limitations that should be considered when interpreting the findings. First, as mentioned, the use of a cross-sectional survey design limits the ability to establish causal relationships. The relationships observed in the study should be interpreted as correlational, rather than causal. For example, while our model posits that social influence increases individuals’ willingness to install a first responder app, it is also plausible that individuals who are already willing to install such an app subsequently develop stronger descriptive and injunctive norm perceptions. To further explore the directional effects and how all constructs within the model influence each other over time, future research should consider using longitudinal or experimental methodologies.

Second, many behavioral determinants were assessed with only 1 or 2 items, rather than more complex, multidimensional scales. This approach was intentional to minimize participant burden and was supported by literature in applied survey research and health psychology, which suggests that single-item measures can be as valid and reliable as their multifaceted counterparts [54]. However, we did not validate the single-item measures in our study, and future research is, therefore, recommended to validate these measures and compare them with multi-item scales to assess whether they perform equally well. This is particularly important, given that some single-item measures (eg, effort expectancy) targeted only specific facets of the constructs rather than capturing them in their entirety.

Third, while the study benefited from a large sample, the use of a web-based survey panel may have introduced self-selection biases. Individuals who join such panels and choose to participate in a CPR-related survey may be more technology-savvy and health-conscious than the general population. This could have led to an over- or underestimation of certain beliefs or attitudes toward the app. For instance, participants may have reported a higher willingness to install the app, as technology-savvy individuals may be more comfortable downloading and using health apps compared to the general public. Future studies should, therefore, consider using alternative or mixed method recruitment strategies, such as random digit dialing or community-based sampling, to validate the results in more diverse and representative populations.

Finally, as there is no existing first responder app for OHCA in Belgium, the study could not measure behavioral intention or actual app use, which is typically measured in studies applying models like UTAUT or the HBM. As a result, we were unable to determine whether participants’ stated willingness would translate into real-world behavior. Additionally, the use of self-reported willingness to install the app may have been subjected to social desirability bias. For example, participants may have overstated their willingness to install the app to appear socially responsible or helpful, especially given the focus on emergency health situations. This intention-behavior gap is well documented in health behavior research and highlights an important limitation of the study. Future research should replicate this work in countries where such apps are already in use to examine whether the identified determinants also predict actual behaviors, such as installation rates, retention over time, and the frequency of CPR interventions triggered by the app.

### Implications and Future Research

The findings of this study have both theoretical and practical implications. Theoretically, it finds its relevance in extending the UTAUT and HBM to altruistic mHealth apps that require active involvement, which has not been done before. Moreover, this study contributes to the field by developing a comprehensive framework that also integrates relevant demographic and health variables, offering a more nuanced understanding of the factors influencing the first responder app for OHCA adoption. Practically, this study was conducted in Belgium, which is a country that currently lacks an established first responder system. As such, the knowledge provided by the study provides practical guidance regarding how to convince citizens in Belgium to participate in the first responder system that health care professionals are currently deploying. Building on this, future research could further enhance these efforts by designing and testing health messages in an experimental setting based on the key determinants of willingness to install a first responder app that we identified in this study. In that way, subsequent studies can refine communication strategies for real-life campaigns. Additionally, it would be valuable to replicate the proposed model in countries where first responder apps for OHCA are already available. Such studies could examine actual adoption behaviors and compare the model’s applicability across different contexts.

This study provides an important first step in mapping the behavioral, demographic, and health-related factors of the Belgian population that influence the willingness to install a first responder app for OHCA. By identifying key behavioral determinants, such as social influence and perceived barriers or benefits, and other sociodemographic and health-related factors, we offer actionable insights that governments can use to shape implementation strategies. For example, campaigns could emphasize the app’s societal benefits and social norms around helping others while also addressing privacy or usability concerns. Additionally, app developers may draw on these insights to design a more accessible and user-friendly first responder app for OHCA. Our findings also point to the need for tailored messaging, as one-size-fits-all communication strategies may not be effective for the entire Belgian population. For instance, older adults may respond differently to a message focused on a certain behavioral determinant than younger adults, suggesting that future research should explore how best to adapt outreach efforts to diverse groups. Future efforts to roll out such technology should be grounded in this type of population-informed research, ensuring that interventions are aligned with what people value and what might hold them back.

## Appendix

Multimedia Appendix 1 Items of the behavioral determinants.

Multimedia Appendix 2 Reliability of multi-item constructs.

Multimedia Appendix 3 Statistics and zero-order correlations.

Multimedia Appendix 4 Indirect relations.

## Abbreviations

CPR: cardiopulmonary resuscitation
CTA: contact tracing app
HBM: health belief model
mHealth: mobile health
OHCA: out-of-hospital cardiac arrest
RMSEA: root mean square error of approximation
RQ: research question
SEM: structural equation model
SES: socioeconomic status
UTAUT: unified theory of acceptance and use of technology

## Data Availability

The datasets generated or analyzed during this study are available in the Open Science Framework repository (osf.io/6k7b5).
