# Supplementary material for: Understanding the Behavioral Determinants of First Responder App Adoption by Integrating Perspectives From the Unified Theory of Acceptance and Use of Technology and Health Belief Model: Cross-Sectional Survey
Source: JMIR Hum Factors. 2025 Sep 9;12:e69934. doi: 10.2196/69934 (PMC12457852; doi:10.2196/69934)
Supplement: Multimedia Appendix 1 [file humanfactors_v12i1e69934_app1.docx]

| Construct | Item |
| --- | --- |
| **UTAUT determinants** |  |
| Performance expectancy | This first responder app is an effective application for saving individuals suffering a cardiac arrest. |
|  | This first responder app would ensure that fewer people die from a cardiac arrest. |
| Effort expectancy | I think it will cost me a lot of time and energy to install this first responder app. |
| Social influence | Most people in my environment would have a positive attitude towards this first responder app. |
|  | Most people in my environment would install this first responder app, if this app would be available in [country blinded]. |
| Facilitating conditions | I have a smartphone at my disposal with access to the internet which allows me to install this first responder app. |
| **HBM determinants** |  |
| Self-efficacy | It seems difficult to install this first responder app. |
|  | I would be able to install this first responder app if I wanted to. |
| Perceived susceptibility | How likely are you to suffer a cardiac arrest? |
|  | How likely is it that an average person of your age will suffer a cardiac arrest? |
| Perceived severity | I believe that if I suffer a cardiac arrest, it could have serious consequences for me. |
|  | I believe that if someone suffers a cardiac arrest, it can have serious consequences for that person. |
| Perceived barriers | I would not install this first responder app because I don’t want to resuscitate someone who’s suffering a cardiac arrest.  I would not install this first responder app because I don’t have a smartphone.  I would not install this first responder app because I don’t find digital technology convenient.  I would not install this first responder app because I’m afraid it will drain my smartphone battery quickly.  I would not install this first responder app because I see few benefits in a first responder app.  I would not install this first responder app because I’m afraid my privacy will not be guaranteed.  I would not install this first responder app because I don’t trust such an app.  I would not install this first responder app because the app would cause me stress.  I would not install this first responder app because the app seems too complex to me.  I would not install this first responder app because I already have too many apps on my smartphone.  I would not install this first responder app because I don’t use my smartphone much.  I would not install this first responder app because I am afraid of getting a notification if someone nearby suffers a cardiac arrest.  I would not install this first responder app because the app is managed by the government.  I would not install this first responder app because I don’t want to share my location with the app. |
| Perceived benefits | I would install this first responder app because it is my duty to help people.  I would install this first responder app because I know people who are struggling with health problems and may need resuscitation one day.  I would install this first responder app because I think it is a useful app.  I would install this first responder app because I want to help people suffering a cardiac arrest.  I would install this first responder app because I know how to resuscitate someone. |
