# Supplementary material for: Understanding the Behavioral Determinants of First Responder App Adoption by Integrating Perspectives From the Unified Theory of Acceptance and Use of Technology and Health Belief Model: Cross-Sectional Survey
Source: JMIR Hum Factors. 2025 Sep 9;12:e69934. doi: 10.2196/69934 (PMC12457852; doi:10.2196/69934)
Supplement: Multimedia Appendix 2 [file humanfactors_v12i1e69934_app2.docx]

| Construct | Number of Items | | Reliability Index | Value |
| --- | --- | --- | --- | --- |
| Performance Expectancy | | 2 | Pearson’s *r* | .75 ^a^ |
| Social Influence | | 2 | Pearson’s *r* | .65 ^a^ |
| Self-efficacy | | 2 | Pearson’s *r* | .45 ^a^ |
| Perceived Susceptibility of an OHCA | | 2 | Pearson’s *r* | .56 ^a^ |
| Perceived Severity of an OHCA | | 2 | Pearson’s *r* | .71 ^a^ |

^a^ *p* < .001
