# Supplementary material for: Understanding the Behavioral Determinants of First Responder App Adoption by Integrating Perspectives From the Unified Theory of Acceptance and Use of Technology and Health Belief Model: Cross-Sectional Survey
Source: JMIR Hum Factors. 2025 Sep 9;12:e69934. doi: 10.2196/69934 (PMC12457852; doi:10.2196/69934)
Supplement: Multimedia Appendix 3 [file humanfactors_v12i1e69934_app3.docx]

|  | ***M*** | ***SD*** | 1 | 2 | | 3 | 4 | 5 | 6 | 7 | 8 | 9 | 10 | 11 | 12 | 13 | 14 | 15 | 16 | 17 | | 18 | | 19 |  |
| --- | --- | --- | --- | --- | --- | --- | --- | --- | --- | --- | --- | --- | --- | --- | --- | --- | --- | --- | --- | --- | --- | --- | --- | --- | --- |
| 1. Gender^d^ | - | - | 1 | | - | - | - | - | - | - | - | - | - | - | - | - | - | - | - | | - | | - | - |  |
| 2. Age | 49.96 | 16.75 | .067^b^ | | 1 | - | - | - | - | - | - | - | - | - | - | - | - | - | - | | - | | - | - |  |
| 3. Education level | 3.84 | 1.01 | -.046^b^ | | -.087^b^ | 1 | - | - | - | - | - | - | - | - | - | - | - | - | - | | - | | - | - |  |
| 4. Medical profession^e^ | - | - | -.104^b^ | | -.100^b^ | .051^b^ | 1 | - | - | - | - | - | - | - | - | - | - | - | - | | - | | - | - |  |
| 5. SES | 6.57 | 1.47 | .003 | | .064^b^ | .404^b^ | .053^b^ | 1 | - | - | - | - | - | - | - | - | - | - | - | | - | | - | - |  |
| 6. General health | 71.11 | 18.05 | .030 | | -.126^b^ | .205^b^ | .036^c^ | .405^b^ | 1 | - | - | - | - | - | - | - | - | - | - | | - | | - | - |  |
| 7. Cardiovascular disease^e^ | - | - | .065^b^ | | .251^b^ | -.062^b^ | -.005 | -.040^c^ | -.234^b^ | 1 | - | - | - | - | - | - | - | - | - | | - | | - | - |  |
| 8. Cardiovascular disease environment^e^ | - | - | -.054^b^ | | -.018 | -.014 | .082^b^ | -.021 | -.093^b^ | .187^b^ | 1 | - | - | - | - | - | - | - | - | | - | | - | - |  |
| 9. CPR training^e^ | - | - | -.014 | | -.184^b^ | .036^c^ | .160^b^ | .070^b^ | .077^b^ | -.041^c^ | .051^b^ | 1 | - | - | - | - | - | - | - | | - | | - | - |  |
| 10. Performance expectancy | 3.9 | .78 | -.001 | | .073^b^ | -.002 | -.024 | .040^c^ | .057^b^ | .000 | -.006 | .039^c^ | 1 | - | - | - | - | - | - | | - | | - | - |  |
| 11. Effort expectancy | 2.06 | 1.05 | -.028 | | .224^b^ | -.140^b^ | .068^b^ | -.096^b^ | -.124^b^ | .111^b^ | .052^b^ | -.124^b^ | -.220^b^ | 1 | - | - | - | - | - | | - | | - | - |  |
| 12. Social influences | 3.28 | .78 | -.002 | | -.051^b^ | .007 | .037^c^ | .053^b^ | .054^b^ | -.014 | .000 | -.029 | .407^b^ | -.087^b^ | 1 | - | - | - | - | | - | | - | - |  |
| 13.Facilitating conditions | 4.04 | 1.08 | -.001 | | -.207^b^ | .132^b^ | -.007 | .118^b^ | .128^b^ | -.075^b^ | .007 | .107^b^ | .224^b^ | -.377^b^ | .136^b^ | 1 | - | - | - | | - | | - | - |  |
| 14. Self-efficacy | 3.02 | .53 | .014 | | -.310^b^ | .156^b^ | -.042^c^ | .099^b^ | .141^b^ | -.119^b^ | -.024 | .134^b^ | .286^b^ | -.660^b^ | .134^b^ | .530 | 1 | - | - | | - | | - | - |  |
| 15. Perceived susceptibility | 2.90 | .64 | .017 | | .421^b^ | -.210^b^ | .002 | -.137^b^ | -.259^b^ | .229^b^ | .087^b^ | -.095^b^ | .048^b^ | .214^b^ | .006 | -.175^b^ | -.233^b^ | 1 | - | | - | | - | - |  |
| 16. Perceived severity | 4.45 | .74 | .004 | | .016 | .179^b^ | -.074^b^ | .106^b^ | .087^b^ | .011 | .009 | .095^b^ | .188^b^ | -.249^b^ | -.011 | .230^b^ | .258^b^ | -.061^b^ | 1 | | - | | - | - |  |
| 17. Perceived barriers | 29.75 | 10.70 | -.053^b^ | | .019 | -.114^b^ | .061^b^ | -.127^b^ | -.151^b^ | .069^b^ | .048^b^ | -.102^b^ | -.434^b^ | .531^b^ | -.299^b^ | -.398^b^ | -.528^b^ | .144^b^ | -.208^b^ | | 1 | | - | - |  |
| 18. Perceived benefits | 18.18 | 3.47 | .013 | | -.044^c^ | -.007 | .133^b^ | .037^c^ | .084^b^ | -.009 | -084^b^ | .273^b^ | .485^b^ | -.164^b^ | .400^b^ | .232^b^ | .251^b^ | .063^b^ | .130^b^ | | -.444^b^ | | 1 | - |  |
| 19. Intention to install a first responder app | 3.64 | 1.03 | .025 | | -.073^c^ | .004 | .053^b^ | .034 | .097^b^ | -.027 | .020 | .113^b^ | .391^b^ | -.221^b^ | .397^b^ | .257^b^ | .280^b^ | .015 | .085^b^ | | -.494^b^ | | .585^b^ | 1 |  |
