# Supplementary material for: Understanding the Behavioral Determinants of First Responder App Adoption by Integrating Perspectives From the Unified Theory of Acceptance and Use of Technology and Health Belief Model: Cross-Sectional Survey
Source: JMIR Hum Factors. 2025 Sep 9;12:e69934. doi: 10.2196/69934 (PMC12457852; doi:10.2196/69934)
Supplement: Multimedia Appendix 4 [file humanfactors_v12i1e69934_app4.docx]

| **Indirect relation** | **Estimate** | **Std. Err** | **Std. Est** |
| --- | --- | --- | --- |
| 1. Age 🡪 performance exp. 🡪 intention | .000 | .000 | .002 |
| 2. Age 🡪 effort exp. 🡪 intention | -.001 | .001 | -.009 |
| 3. Age 🡪 facilitating cond. 🡪 intention | -.001^**^ | .000 | -.012^**^ |
| 4. Age 🡪 social influences 🡪 intention | -.001^**^ | .000 | -.012^**^ |
| 5. Age 🡪 self-efficacy 🡪 intention | .002 | .002 | .032 |
| 6. Age 🡪 perceived suscept. 🡪 intention | .002^**^ | .001 | .036^**^ |
| 7. Age 🡪 perceived sever. 🡪 intention | -.000 | .000 | -.001 |
| 8. Age 🡪 perceived barriers 🡪 intention | .000 | .000 | .004 |
| 9. Age 🡪 perceived benefits 🡪 intention | .000 | .000 | .005 |
| 10. Gender 🡪 performance exp. 🡪 intention | -.001 | .001 | -.000 |
| 11. Gender 🡪 effort exp. 🡪 intention | .004 | .004 | .002 |
| 12. Gender 🡪 facilitating cond. 🡪 intention | .002 | .002 | .001 |
| 13. Gender 🡪 social influences 🡪 intention | .002 | .006 | .001 |
| 14. Gender 🡪 self-efficacy 🡪 intention | -.010 | .009 | -.005 |
| 15. Gender 🡪 perceived suscept. 🡪 intention | .002 | .002 | .001 |
| 16. Gender 🡪 perceived sever. 🡪 intention | .000 | .001 | .000 |
| 17. Gender 🡪 perceived barriers 🡪 intention | .029^**^ | .011 | .014^**^ |
| 18. Gender 🡪 perceived benefits 🡪 intention | .018 | .013 | .009 |
| 19. SES 🡪 performance exp. 🡪 intention | .000 | .000 | .000 |
| 20. SES 🡪 effort exp. 🡪 intention | .002 | .002 | .002 |
| 21. SES 🡪 facilitating cond. 🡪 intention | .003 | .001 | .005 |
| 22. SES 🡪 social influences 🡪 intention | .008^**^ | .003 | .011 |
| 23. SES 🡪 self-efficacy 🡪 intention | -.003 | .003 | -.005 |
| 24. SES 🡪 perceived suscept. 🡪 intention | -.002 | .001 | -.003 |
| 25. SES 🡪 perceived sever. 🡪 intention | -.000 | .000 | -.000 |
| 26. SES 🡪 perceived barriers 🡪 intention | .011 | .004 | .015 |
| 27. SES 🡪 perceived benefits 🡪 intention | .001 | .005 | .001 |
| 28. Education level 🡪 performance exp. 🡪 intention | -.000 | .001 | -.000 |
| 29. Education level 🡪 effort exp. 🡪 intention | .004 | .005 | .004 |
| 30. Education level 🡪 facilitating cond. 🡪 intention | .005 | .002 | .005 |
| 31. Education level 🡪 social influences 🡪 intention | -.004 | .003 | -.003 |
| 32. Education level 🡪 self-efficacy 🡪 intention | -.013 | .011 | -.013 |
| 33. Education level 🡪 perceived suscept. 🡪 intention | -.007^**^ | .003 | -.007^**^ |
| 34. Education level 🡪 perceived sever. 🡪 intention | -.004 | .003 | -.004 |
| 35. Education level 🡪 perceived barriers 🡪 intention | .020^**^ | .006 | .020^**^ |
| 36. Education level 🡪 perceived benefits 🡪 intention | -.013 | .007 | -.012 |
| 37. Medical prof. 🡪 performance exp. 🡪 intention | -.003 | .003 | -.001 |
| 38. Medical prof. 🡪 effort exp. 🡪 intention | -.016 | .018 | -.005 |
| 39. Medical prof. 🡪 facilitating cond. 🡪 intention | -.010 | .005 | -.003 |
| 40. Medical prof. 🡪 social influences 🡪 intention | .018 | .011 | .005 |
| 41. Medical prof. 🡪 self-efficacy 🡪 intention | .042 | .037 | .012 |
| 42. Medical prof. 🡪 perceived suscept. 🡪 intention | .016 | .006 | .005 |
| 43. Medical prof. 🡪 perceived sever. 🡪 intention | .008 | .006 | .002 |
| 44. Medical prof. 🡪 perceived barriers 🡪 intention | -.081^***^ | .019 | -.024^***^ |
| 45. Medical prof. 🡪 perceived benefits 🡪 intention | .087^***^ | .022 | .026^***^ |
| 46. Health 🡪 performance exp. 🡪 intention | .000 | .000 | .002 |
| 47. Health 🡪 effort exp. 🡪 intention | .000 | .000 | .002 |
| 48. Health 🡪 facilitating cond. 🡪 intention | .000 | .000 | .003 |
| 49. Health 🡪 social influences 🡪 intention | .000 | .000 | .006 |
| 50. Health 🡪 self-efficacy 🡪 intention | -.000 | .000 | -.006 |
| 51. Health 🡪 perceived suscept. 🡪 intention | -.000^**^ | .000 | -.006^**^ |
| 52. Health 🡪 perceived sever. 🡪 intention | -.000 | .000 | -.001 |
| 53. Health 🡪 perceived barriers 🡪 intention | .002^***^ | .000 | .030^***^ |
| 54. Health 🡪 perceived benefits 🡪 intention | .002 | .000 | .028^***^ |
| 55. Cv. disease 🡪 performance exp. 🡪 intention | .000 | .002 | .000 |
| 56. Cv. disease 🡪 effort exp. 🡪 intention | -.004 | .005 | -.002 |
| 57. Cv. disease 🡪 facilitating cond. 🡪 intention | -.001 | .003 | .000 |
| 58. Cv. disease 🡪 social influences 🡪 intention | .003 | .009 | .001 |
| 59. Cv. disease 🡪 self-efficacy 🡪 intention | .008 | .009 | .003 |
| 60. Cv. disease 🡪 perceived suscept. 🡪 intention | .005 | .003 | .002 |
| 61. Cv. disease 🡪 perceived sever. 🡪 intention | -.002 | .002 | -.001 |
| 62. Cv. disease 🡪 perceived barriers 🡪 intention | -.033 | .016 | -.012 |
| 63. Cv. disease 🡪 perceived benefits 🡪 intention | -.007 | .019 | -.002 |
| 64. CPR training 🡪 performance exp. 🡪 intention | .003 | .003 | .001 |
| 65. CPR training 🡪 effort exp. 🡪 intention | .009 | .010 | .004 |
| 66. CPR training 🡪 facilitating cond. 🡪 intention | .009 | .004 | .005 |
| 67. CPR training 🡪 social influences 🡪 intention | -.016 | .007 | -.008 |
| 68. CPR training 🡪 self-efficacy 🡪 intention | -.021 | .019 | -.010 |
| 69. CPR training 🡪 perceived suscept. 🡪 intention | -.004 | .002 | -.002 |
| 70. CPR training 🡪 perceived sever. 🡪 intention | -.006 | .004 | -.003 |
| 71. CPR training 🡪 perceived barriers 🡪 intention | .065^***^ | .012 | .031^***^ |
| 72. CPR training 🡪 perceived benefits 🡪 intention | .202^***^ | .017 | .096^***^ |
| 73. Cv. disease environ. 🡪 performance exp. 🡪 intention | .000 | .001 | .000 |
| 74. Cv. disease environ. 🡪 effort exp. 🡪 intention | -.004 | .005 | -.002 |
| 75. Cv. disease environ. 🡪 facilitating cond. 🡪 intention | .002 | .003 | .001 |
| 76. Cv. disease environ. 🡪 social influences 🡪 intention | .000 | .007 | .000 |
| 77. Cv. disease environ. 🡪 self-efficacy 🡪 intention | .006 | .007 | .003 |
| 78. Cv. disease environ. 🡪 perceived suscept. 🡪 intention | .007 | .003 | .003 |
| 79. Cv. disease environ. 🡪 perceived sever. 🡪 intention | -.001 | .001 | .000 |
| 80. Cv. disease environ. 🡪 perceived barriers 🡪 intention | -.019 | .012 | -.009 |
| 81. Cv. disease environ. 🡪 perceived benefits 🡪 intention | .059^***^ | -.026 | .026^***^ |

*Note*. *** *p* < .001; ** *p* < .01; * *p* < .05.

Gender is coded: woman = 1, man = 2, other = 3. Medical prof., Cv. Disease, CPR training and Cv. Disease environ. are coded: yes = 1 and no = 0
